# Genome-wide identification and functional characterization of the *Camelina sativa WRKY* gene family in response to abiotic stress

**DOI:** 10.1186/s12864-020-07189-3

**Published:** 2020-11-11

**Authors:** Yanan Song, Hongli Cui, Ying Shi, Jinai Xue, Chunli Ji, Chunhui Zhang, Lixia Yuan, Runzhi Li

**Affiliations:** 1 Institute of Molecular Agriculture and Bioenergy, Shanxi Agricultural University, Jinzhong, Shanxi China; 2 College of Biological Science and Technology, Jinzhong University, Jinzhong, Shanxi China

**Keywords:** Camelina (*Camelina sativa* (L.) Crantz), WRKY transcriptional factors, Genome-wide characterization, Expression profiles, Function analysis, Abiotic stress

## Abstract

**Background:**

WRKY transcription factors are a superfamily of regulators involved in diverse biological processes and stress responses in plants. However, there is limited knowledge about the WRKY family in camelina (*Camelina sativa*), an important Brassicaceae oil crop with strong tolerance for various stresses. Here, a genome-wide characterization of WRKY proteins is performed to examine their gene structures, phylogenetics, expression, conserved motif organizations, and functional annotation to identify candidate WRKYs that mediate stress resistance regulation in camelinas.

**Results:**

A total of 242 CsWRKY proteins encoded by 224 gene loci distributed unevenly over the chromosomes were identified, and they were classified into three groups by phylogenetic analysis according to their WRKY domains and zinc finger motifs. The 15 *CsWRKY* gene loci generated 33 spliced variants. Orthologous *WRKY* gene pairs were identified, with 173 pairs in the *C. sativa* and *Arabidopsis* genomes as well as 282 pairs in the *C. sativa* and *B. napus* genomes, respectively. A total of 137 segmental duplication events were observed, but there was no tandem duplication in the camelina genome. Ten major conserved motifs were examined, with WRKYGQK being the most conserved, and several variants were present in many CsWRKYs. Expression analysis revealed that 50% more *CsWRKY* genes were expressed constitutively, and a set of them displayed tissue-specific expression. Notably, 11 *CsWRKY* genes exhibited significant expression changes in seedlings under cold, salt, and drought stresses, showing a preferentially inducible expression pattern in response to the stress.

**Conclusions:**

The present article describes a detailed analysis of the *CsWRKY* gene family and its expression profiles in 12 tissues and under several stress conditions. Segmental duplication is the major force underlying the broad expansion of this gene family, and a strong purifying pressure occurred for CsWRKY proteins during their evolution. CsWRKY proteins play important roles in plant development, with differential functions in different tissues. Exceptionally, eleven CsWRKYs, particularly five alternative spliced isoforms, were found to be the possible key players in mediating plant responses to various stresses. Overall, our results provide a foundation for understanding the roles of CsWRKYs and the precise mechanism through which CsWRKYs regulate high stress resistance as well as the development of stress tolerance cultivars among *Cruciferae* crops.

**Supplementary Information:**

The online version contains supplementary material available at 10.1186/s12864-020-07189-3.

## Background

The camelina (*Camelina sativa* (L.) Crantz), a dicotyledonous plant, belongs to the *Cruciferae* family. As a “low-input and environment-friendly” oil crop grown around the world, the camelina has been planted in many countries in Europe and the Middle East in addition to China [1]. Compared with other commercialized oil crops, *C. sativa* has several prominent agronomic traits, such as a short life cycle (80–100 days), strong tolerance to abiotic stresses (salt, drought, cold, etc.), and high resistance to common pests and diseases that infect many cruciferous crops [2–5]. Camelina seeds accumulate high levels of oil (36% ~ 47%) and protein (30%) as well as a variety of natural active ingredients. Its seed oil contains 90% unsaturated fatty acids, of which omega-3 fatty acids constitute 40% or more of the total. Given such high nutritional and functional value towards improving human immunity and protection against various diseases [5, 6], *C. sativa* provides the sustainable feedstock for the commercial production of food, feed, biofuel (e.g., aviation fuel and biodiesel), and other high-value industrial products [7]. Numerous studies have focused on camelina seed yields and oil quality [8, 9]. For example, the over-expression of microRNA167A in *C. sativa* seeds reduced the α-linolenic acid content and increased the seed size [10]. The expression of diacylglycerol acyltransferase 1 and glycerol-3-phosphate dehydrogenase increased the seed oil yields in *C. sativa* [11]. The seed-specific suppression of ADP-glucose pyrophosphorylase enhanced the seed size and weight in *C. sativa* [12]. However, there are few reports on the molecular mechanism underlying the high tolerance to various stresses found in *C. sativa*.

Abiotic and biotic stresses seriously affect agricultural production, leading to reductions in the crop yield and quality [4]. To adapt to diverse stresses, plants have evolved favourable strategies such as metabolic reconstruction, cell-tissue remodelling, and gene expression reprogramming. Transcriptional factors (TFs) can bind to *cis* elements or interact with other regulatory factors to regulate the expression of downstream defence-related genes [13]. Increasing numbers of reports show that a number of different TFs play a significant regulatory role in plant stress responses, with TFs including bHLH [14], MYB [15], bZIP [16], NAC [17], WRKY [18], and others. Among these proteins, WRKY proteins (WRKYs) are one of the largest TF families, with complex biological functions and specificity to many plant species ranging from single-celled algae to higher plants. For instance, in *Arabidopsis thaliana*, the overexpression of AtWRKY50 promoted the production of sinapic derivatives [19]. AtWRKY46, 54, and 70 have important effects in terms of activating the expression of brassinosteroid-mediated genes and restraining the drought gene response [20]. OsWRKY47 positively regulates both the yield and drought tolerance of rice [21]. TaWRKY33 significantly increased the wheat drought tolerance [22]. PtrWRKY18 and PtrWRKY35 enhanced resistance to *Melampsora* in *Populus* [23].

All known WRKYs contain one or two unique DNA binding domains consisting of approximately 60 amino acids (aa) that are characterized by a highly conserved WRKYGQK sequence (designated as the WRKY domain) at the N-terminus, followed by a C2H2 zinc-finger-like motif (C-X4–5C-X22–23-H-X1-H or C-X7C-X23-H-X1-C) at the C-terminus [13]. The WRKY domain specifically binds to the consensus W-box (a *cis*-acting element with the core sequence TGAC) in promoters of the target genes [24]. According to the number of WRKY domains and the type of zinc-finger-like structure, WRKY proteins are generally classified into three primary groups (I-III). Group I WRKY proteins contain two WRKY domains and the zinc finger motif of C-X4-C-X22–23-H-X1-H, whereas Groups II and III have only one WRKY domain, with group II proteins sharing the same zinc finger motif as group I and group III proteins and group III bearing the unique zinc finger motif of C-X4–5-C-X23–24-H-X1-H [25]. Group II WRKYs can be further divided into several distinct subgroups (IIa-e) based on their phylogenetic relationship. In addition, some special resistance protein (R-protein) WRKYs were found in several plant species, with three R-protein WRKYs in *Arabidopsis* (AtWRKY16, AtWRKY19 and AtWRKY52) and one R-protein WRKY in soybeans (GmWRKY1) [26] and pineapples (AcWRKY23) [27]. The R-protein WRKY may further enhance the signal diversity and even shorten the speed of signal transmission with other components of signalling pathways.

Since the first WRKY gene (*SPF1*) was examined in sweet potatoes (*Ipomoea batatas*) [28], numerous members of the WRKY gene family have been identified in a variety of plant species, including 72 from *Arabidopsis*, 97 from wild rice, 83 from tomatoes, and 119 from corn [24, 29, 30] (for the details, please see Additional file 1: Table S1). Increasing numbers of reports demonstrate that the WRKY proteins have important functions in plant defence against various biotic and abiotic stresses. For example, GmWRKY45 overexpression had a positive effect on the *Arabidopsis* response to phosphorus and salinity stress, and it also resulted in changes in fertility [31]. The expressions of *GmWRKY92, 144* and *165* were highly upregulated during soybean responses to salinity stress [32]. VvWRKY30 from grapes (*Vitis vinifera* L.) was confirmed to confer tolerance to salt stress [33]. Based on these previous findings, we hypothesize that WRKY TFs may mediate the regulation of stress resistance in *C. sativa* despite no genome-wide detection of this gene family being conducted in this oil crop. Thus, studying the WRKY gene family would shed light on the molecular mechanism underlying strong stress tolerance in camelina, providing valuable information for the genetic improvement of this oilseed and other crops.

Therefore, the current study was conducted to perform a genome-wide characterization of WRKY family members in *C. sativa.* A total of 242 CsWRKY proteins were identified from the camelina genome. Subsequently, a comprehensive analysis was employed to examine their physicochemical properties and conserved motifs, the chromosomal locations and duplications of their genes as well as their intron-exon structures. To reveal their evolutionary relationships, a phylogenetic tree was constructed through the combined use of WRKY proteins identified in *Arabidopsis thaliana* from the *Cruciferae* family. Moreover, the expression profiles of the *CsWRKY* genes were extensively detected using publicly available RNA-Seq data, and a set of selected *CsWRKY* gene transcripts were also verified by quantitative real-time RT-PCR (qRT-PCR) in camelina seedlings under stress conditions. Notably, 33 alternative splicing events were identified among the *CsWRKY* genes, showing that different spliced variants derived from the same *CsWRKY* gene were expressed in varying ratios in response to different stresses. Our study offers new scientific resources for understanding the biological roles of CsWRKYs, particularly in plant resistance to various stresses.

## Results

### Identification of 242 WRKY family members in *Camelina sativa*

To identify the WRKY proteins encoded in the camelina genome, all 72 *Arabidopsis* AtWRKY protein sequences were used as queries to search the publicly available genome sequences of *C. sativa* by BLAST (Basic Local Alignment Search Tool), and then they were examined using an HMM (hidden Markov model) with the WRKY-domain (PF03106). A total of 243 putative WRKYs were initially obtained from *Camelina sativa.* One predicted WRKY protein (XP_019082498.1) was removed due to the incomplete WRKY domain. The remaining 242 WRKYs (see Additional file 2: Table S2) were renamed from CsWRKY1 to CsWRKY242, and their characteristics were further specified, including their gene locus ID, gene start and end position in the chromosomes, member classification, protein sequence length (SL), molecular weight (MW) and isoelectric point (pI). The length of the CsWRKY proteins ranged from 136 (CsWRKY136) to 1699 amino acids (CsWRKY47), with an average of 371 amino acids. Their MWs ranged from 15.98 kDa (CsWRKY136) to 190.70 kDa (PvWRKY34), with an average of 41.33 kDa. The pIs of the CsWRKYs ranged from 4.13 (CsWRKY204) to 10.47 (CsWRKY107), with an average of 7.38 (Additional file 2: Table S2).

Compared with the numbers of WRKY family members reported in other plant species, the CsWRKY family with 224 gene loci coding for 242 proteins is one of the largest WRKY families, and it is slightly smaller than that of *Brassica napus* (287 WRKY genes) (Additional file 1: Table S1), indicating that this TF family was extensively expanded in *C. sativa* during its evolutionary process.

### Alternative splicing (AS) occurs among 15 *CsWRKY* genes, producing 33 spliced ORFs

Alternative splicing (AS) is an important post-transcriptional regulatory mechanism that causes one gene locus to generate two or more ORFs (open reading frame) that differ in their initiation and termination sites as well as splice donor and acceptor sites. These alternative ORFs encode different protein variants from the same gene locus. In this study, we found that 15 *CsWRKY* genes have different types of AS events such as exon skipping and intron retention, producing 33 spliced variants of CsWRKY (see Additional file 3: Table S3, Additional file 8: Figure S1). All the splice variants were completely matched by the corresponding mRNAs identified in camelina. For example, *Csa06g039950* had two spliced variants (CsWRKY21 and 22) while *Csa10g016180* generated three spliced variants (CsWRKY41, 35, and 38). These results indicate that alternative splicing might act in regulating the function of WRKY family members.

### Multiple sequence alignment and phylogenetic analysis of CsWRKY proteins

**Fig. 1:**
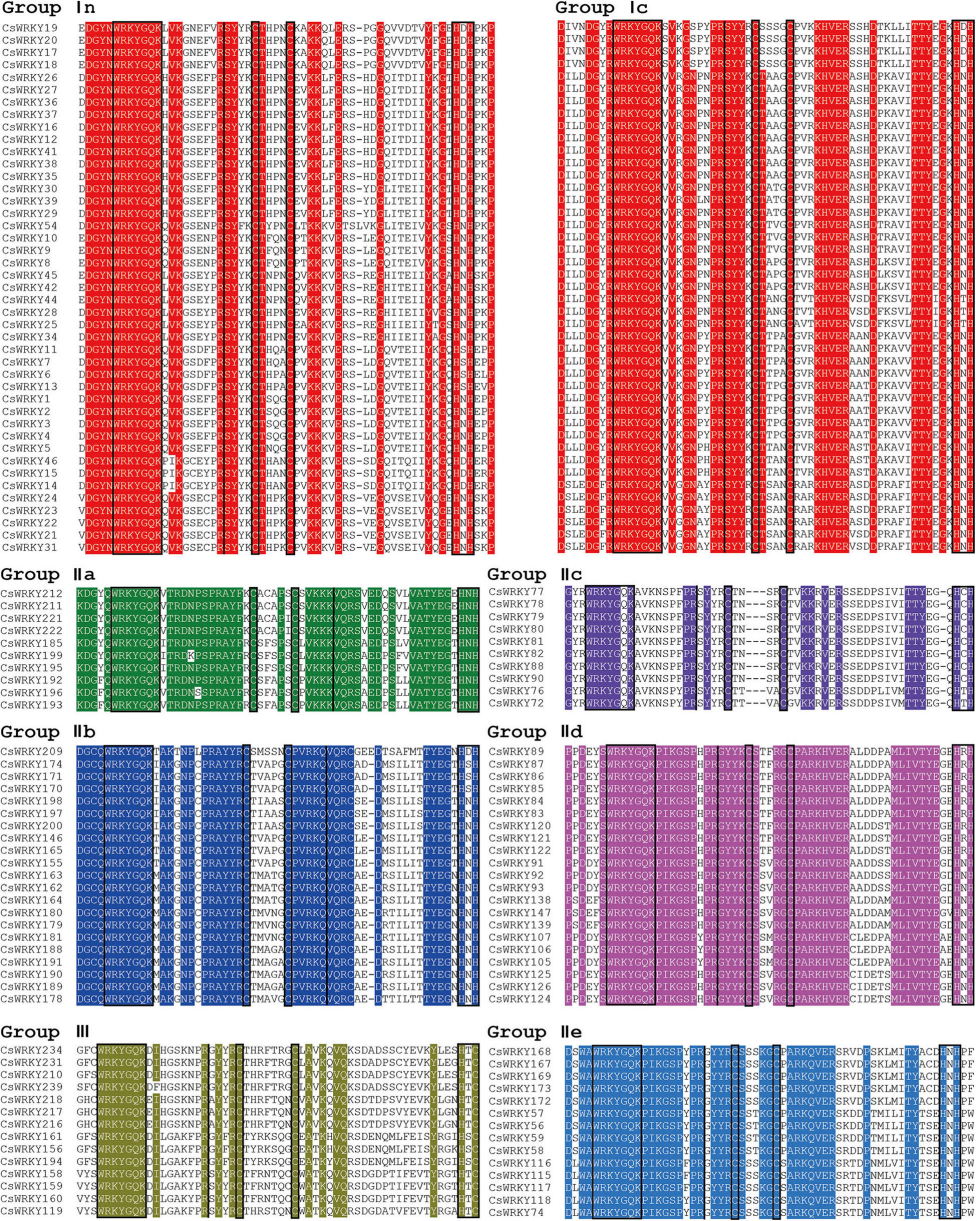
Multiple sequence alignment of the WRKY domain from CsWRKYs. Black box indicated the conserved WRKY amino acid sequence and zinc-finger domain. Black line indicated the position of the conserved PR intron and VQR intron

**Fig. 2:**
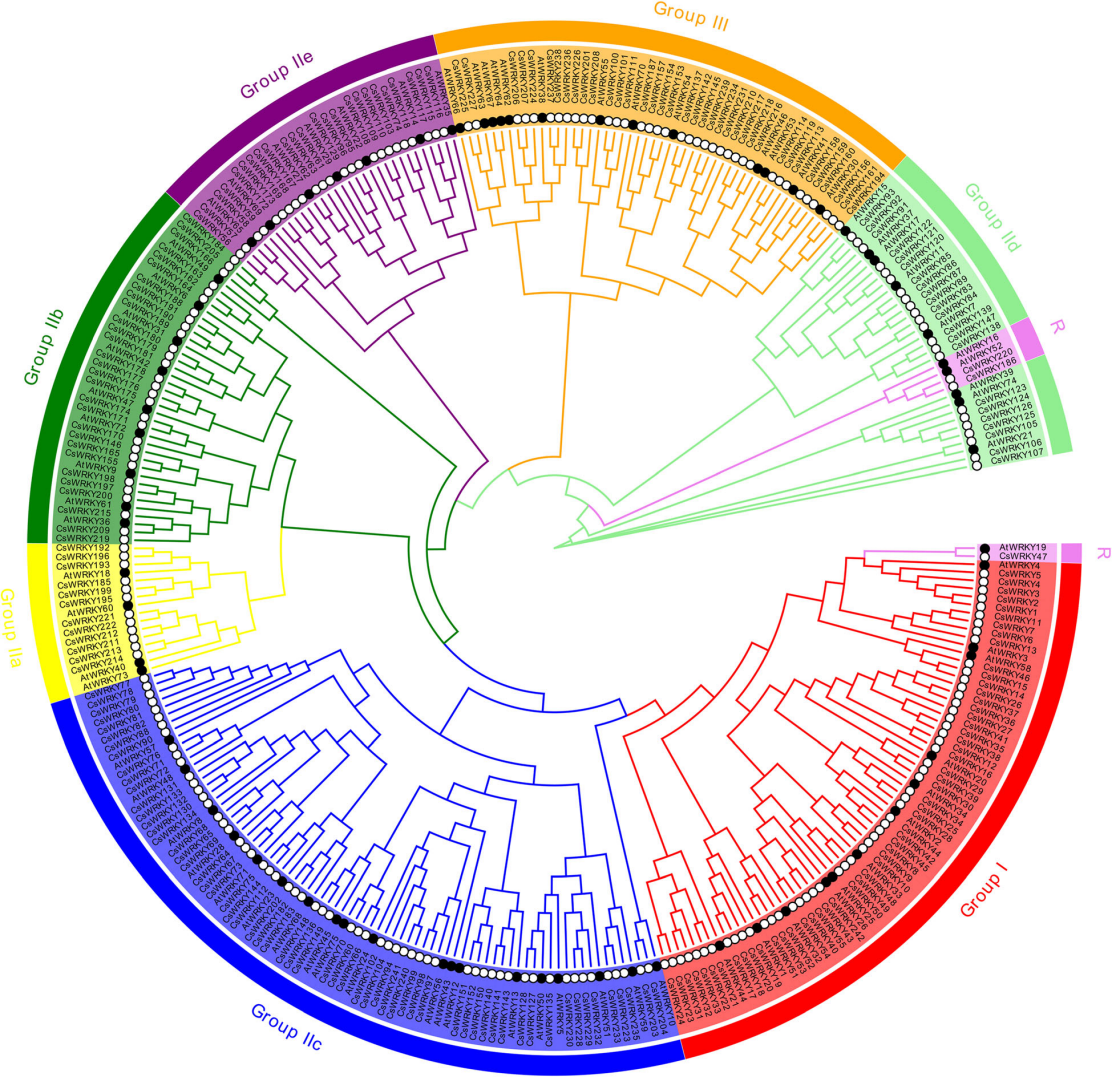
Comparative phylogenetic tree showed the domains relationship of CsWRKYs and AtWRKYs. The identified CsWRKYs (black hollow circle) and scattered AtWRKYs (black solid circle) were clustered to the different groups in phylogenetic tree. The various colors represent the different groups (or subgroups) of CsWRKY domains

To investigate the evolutionary relationships among CsWRKY proteins, a multiple protein sequence alignment of all 242 CsWRKYs was conducted using ClustalW with Bioedit software. The alignment result of the randomly-selected CsWRKYs is shown in Fig. 1 (for details, see Additional file 9: Figure S2). Subsequently, based on the highly conserved WRKY domains of 242 CsWRKYs and 72 AtWRKYs, a phylogenetic tree was built with MEGA7.0 software using the neighbour-joining method (Fig. 2).

The WRKYGQK heptapeptide is considered as the signature of WRKY proteins. As shown in Fig. 1, however, WRKY domain amino acid sequence variants were detected among 11 CsWRKYs (all in group IIc), although WRKYGQK was the most common variant present in 231 CsWRKYs. For the WRKYGQK sequence, most variations involved Q to K substitutions, with a few cases from Q to other amino acids. For example, the variant WRKYGXK sequence (X is any amino acid) was detected in CsWRKY202, but the variant WRKYGKK sequence was present in 10 CsWRKYs including CsWRKY127, 128, 135, 223, 228, 229, 230, 232, 233 and 235. All these variants occurred in group IIc, showing that the WRKYGQK sequence in subgroup IIc WRKY proteins are prone to mutation, similar to the WRKY families from peanuts [34] and soybeans [32].

According to the constructed phylogenetic tree containing CsWRKYs and AtWRKYs (Fig. 2), the CsWRKYs were classified into three primary groups (groups I, II and III), with 56 CsWRKYs in group I, 149 CsWRKYs in group II, and 37 CsWRKYs in group III. Moreover, the CsWRKYs in group II were further classified into five subgroups (groups IIa, IIb, IIc, IId and IIe) containing 12, 26, 63, 24, and 24 members of CsWRKYs, respectively. Notably, among all these groups or subgroups, most members of the CsWRKYs were present in subgroup IIc, which is similar to AtWRKY proteins. The combined description above indicates that WRKYGQK sequence variants were only present in subgroup IIc, suggesting that subgroup IIc WRKY proteins might be involved in a variety of biological functions.

Of the 242 CsWRKYs found here, 56 CsWRKYs in group I had two conserved WRKY domains located in the N- and C-termini of the protein as well as the zinc-finger domains of C-X4-C-X22–23-H-X1-H. A total of 186 CsWRKYs in groups II and III contained one WRKY domain. The 149 CsWRKYs in group II had the zinc-finger domain C-X4–5-C-X23–24-H-X1-H while 37 CsWRKYs in group III had the zinc-finger domain C-X7-C-X23-H-X1-C. It is notable that based on the multiple sequence alignment, either one or two more mismatched amino acids were detected within the conserved regions of 144 CsWRKY proteins, including CsWRKY100, 101, 111, 186, 201, 208, 226, 240, 241, and 242. These annotation errors might be caused by genomic sequencing or gene prediction software.

As shown in Fig. 1, each WRKY domain had at least one conserved intron structure except the N-terminal WRKY domain of the group I WRKYs. The intron insertion position was also rather conserved. Two primary types of intron structures were present in the conserved regions of the CsWRKY genes, much like those conserved in AtWRKYs [35]. One of the introns is a PR intron, which was spliced at the codon of R amino acid. The other is the VQR intron. The PR intron is located in the WRKY domains of CsWRKY genes in group III and subgroups Ic and IIc–e. However, the VQR intron is present within the zinc-finger structure (C-X4–5-C-X5-VQR-X18–19-H-X1-H) in groups IIa and IIb.

A number of WRKY proteins contain both R-protein conserved domains and the typical WRKY domain, and thus they are named R-protein WRKYs, which are one of the most significant features of the WRKY proteins in flowering plants [25]. For example, *Arabidopsis* had three R protein-WRKYs (AtWRKY16, AtWRKY19 and AtWRKY52) [26]. Here, the phylogenetic analysis showed that AtWRKY19 and CsWRKY47 were clustered closely in group I while AtWRKY16, AtWRKY52, CsWRKY186 and CsWRKY220 were highly classified together into subgroup IId. This result indicated that CsWRKY47, CsWRKY186 and CsWRKY220 may belong to the R-protein WRKYs. A further protein architecture analysis identified three CsWRKY proteins as the R-protein WRKYs. CsWRKY47 contained the typical domain PAH-WRKY (1-N terminus)-WRKY (1-C terminus)-NB-ARC and the protein kinase domain at the C-terminal end of the protein. CsWRKY186 had the typical domain NB-ARC-LRR-WRKY. CsWRKY220 had the TIR-NB-ARC-LRR-WRKY domain. The three R-protein CsWRKYs might be involved in camelina responses to biotic and abiotic stress.

### Ten conserved motifs were detected in CsWRKY proteins

**Fig. 3:**
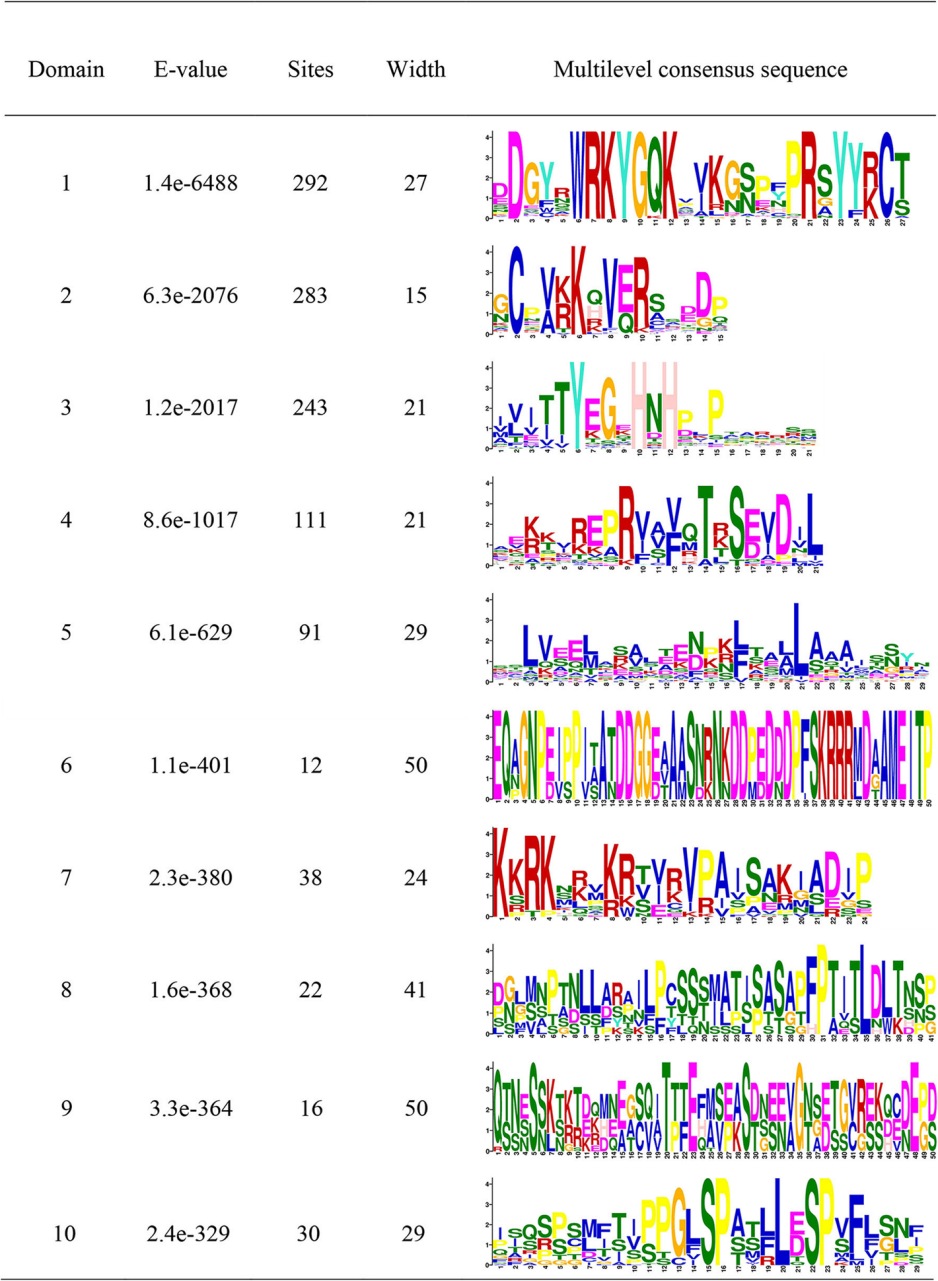
Details of 10 Motifs of CsWRKYs

To gain insight into the functional regions of CsWRKY proteins, the MEME (Multiple Em for Motif Elicitation) program was employed to reveal the conserved motifs among 242 CsWRKY proteins. A total of 10 conserved motifs were identified, namely Motifs 1–10 (Fig. 3). These 10 conserved motifs are indicated with coloured boxes according to their scale (Additional file 10: Figure S3), with sizes ranging from 15 to 50 aa residues in width (Fig. 3). Among these individuals, motif 1 was found to encode the conserved WRKY domain and motifs 2 and 3 were found to encode the conserved zinc finger structure. One or two WRKY motifs were detected in all the CsWRKYs as described in the sequence alignment (Fig. 1). In addition to the conserved WRKY motif, other conserved motifs (motifs 4–10) were also predicted to exist among the CsWRKY proteins. Each CsWRKY protein had at least three conserved motifs, with the maximum number being seven conserved motifs for several CsWRKYs. The distributions of the conserved motifs were diverse among the different CsWRKY groups. For example, group I CsWRKYs had nine motifs (motifs 1, 2, 3, 4, 5, 6, 8, 9 and 10), with each having at least two of the motif 1 and one zinc finger (motifs 2 and 3) as well as motifs 6 and 9, which were unique to the CsWRKY members in group I. Motif 10 only appeared in group I and subgroup IIc. Motifs 4 and 8 were present in group I and subgroups IIb and IIc. Motif 7 was present in subgroups IId and IIe and group III. Motif 5 was only absent from subgroups IIc and IIe. However, all the CsWRKY members of group IIa contained two conserved Motif 5 s. On the whole, the motif analysis of CsWRKYs showed that every group or subgroup of CsWRKYs had similar motif compositions corresponding to the grouping given by the phylogenetic tree analysis.

### *CsWRKY* genes were unevenly distributed on chromosomes

**Fig. 4:**
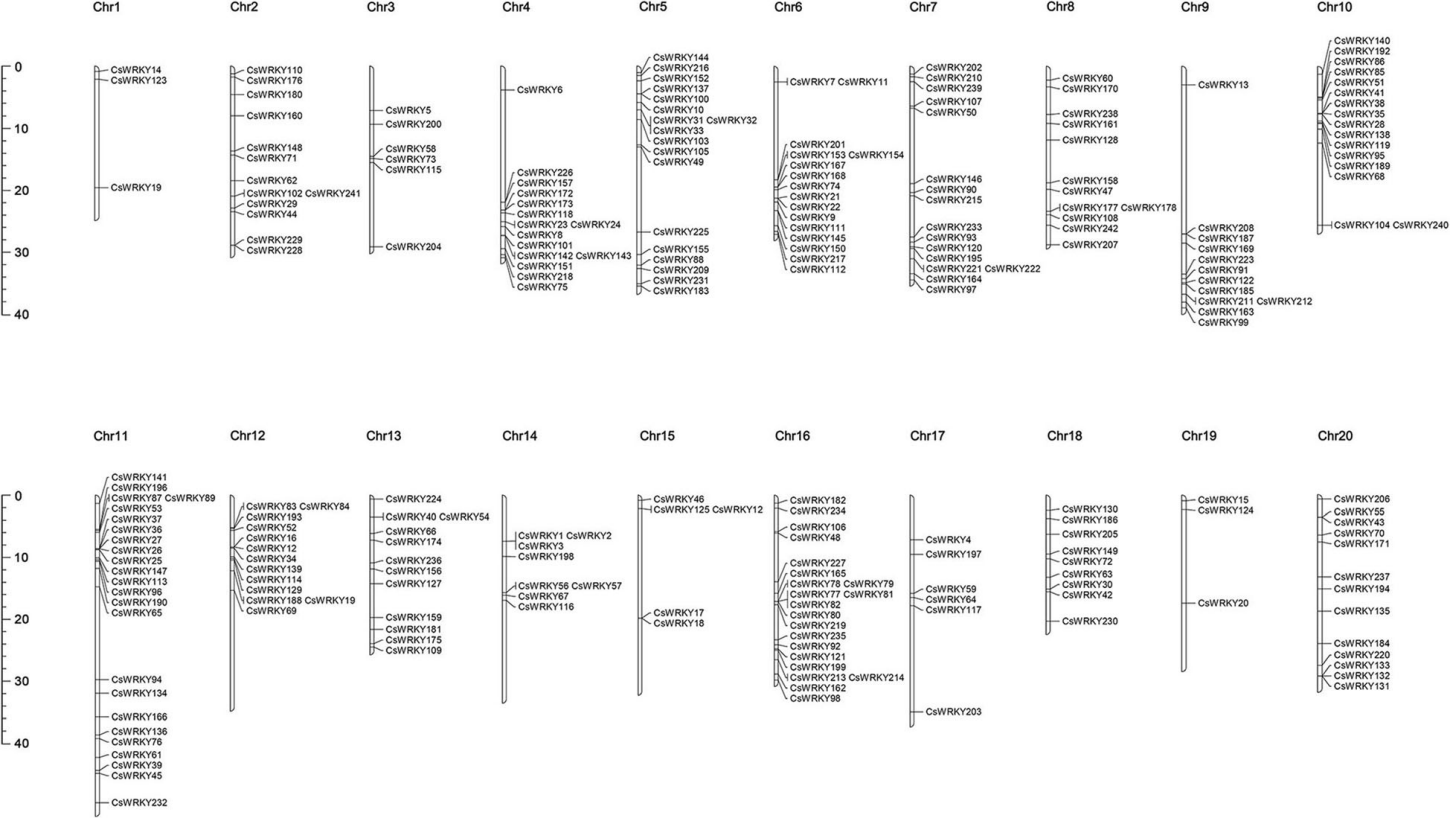
Chromosomal distribution of CsWRKY genes. The position of every CsWRKY gene can be determined using the left scale

To determine the genomic distribution of the *CsWRKY* genes, all 242 identified CsWRKY mRNAs/ORFs were mapped onto their corresponding chromosome by BLAST against the released genome for *C. sativa*. A total of 224 CsWRKY gene loci were distributed across all *C. sativa* chromosomes visualized by MapChart (Chr1-Chr20, Fig. 4), with 15 CsWRKY gene loci generating 33 alternatively spliced variants of CsWRKY ORFs (Additional file 3: Table S3) (for details, see Additional file 8: Figure S1). However, the distribution and density of the WRKY genes on each chromosome were uneven. For example, the minimal number of *CsWRK*Y genes (three loci) were located on chromosomes 1, 15 and 19, whereas the largest number of *CsWRK*Y genes (20 loci) were detected on chromosome 11, which accounted for 8.3% of all the *CsWRKY* genes. Moreover, the same numbers of *CsWRKY* genes (11 loci) were present on chromosomes 2, 8, 9 and 13. One exception is that *CsWRKY179* was not mapped to any chromosome since it is present on a scaffold region. Interestingly, a few regions with a higher density of CsWRKY genes were observed on some chromosomes, such as chromosomes 10, 11, and 16, suggesting that there might be *WRKY* gene hot spots in the camelina genome.

### *CsWRKY* gene family experienced 137 segmental duplication events, with high synteny with *WRKYs* from *Arabidopsis* and *Brassica rapa*

**Fig. 5:**
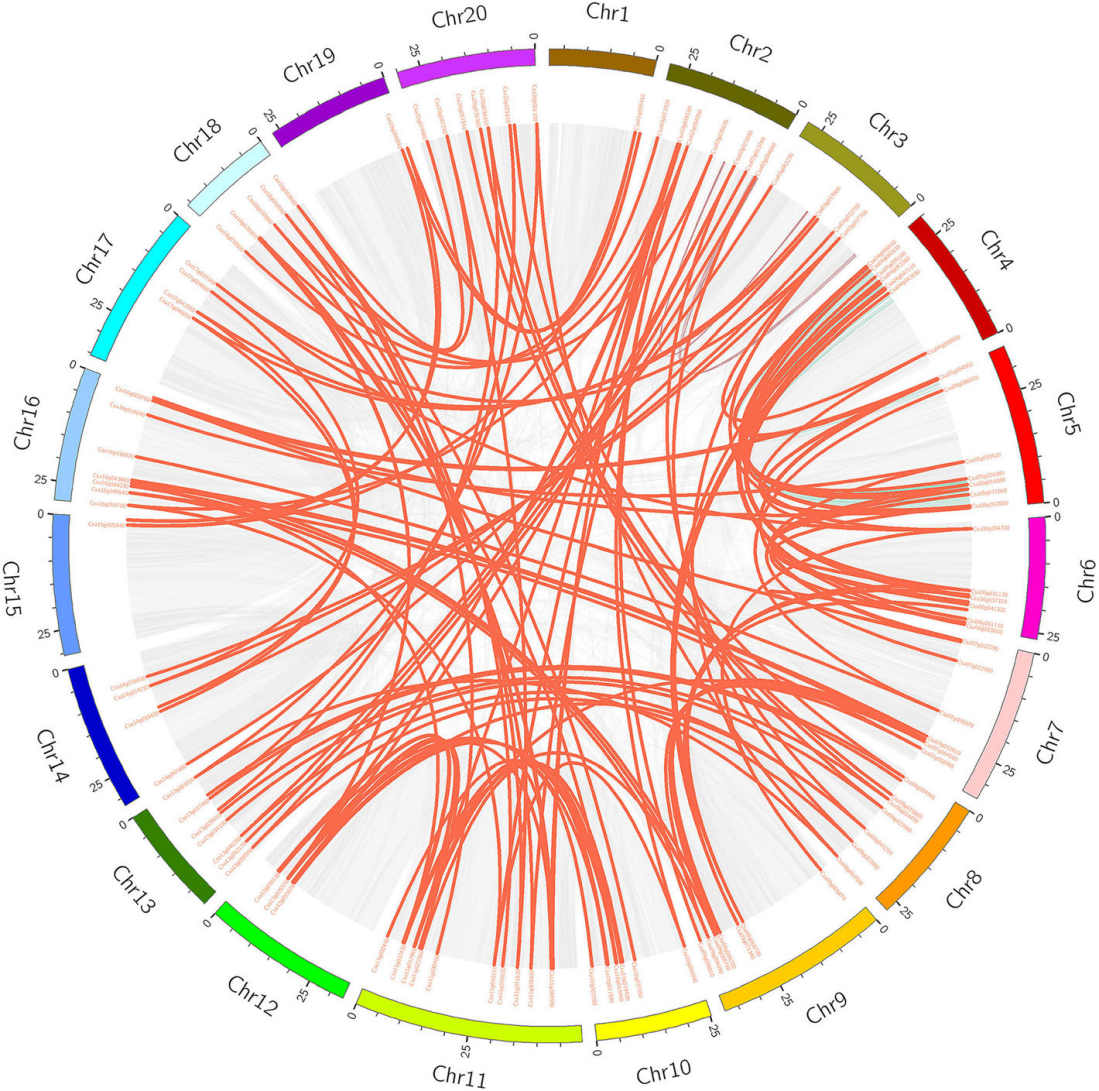
Synteny analysis of interchromosomal relationships of CsWRKY genes. All gene pairs and CsWRKY gene pairs in the *C. sativa* genome were indicated by gray lines and red lines respectively

As described above, the *CsWRKY* gene family expanded greatly compared to other plant *WRKY* families, ranking as the second most common in the quantity of *WRKY* members tested so far. To elucidate the mechanism underlying WRKY gene family expansion in *C. sativa*, BLASTp and MCScanX (Multiple Collinearity Scan toolkit) were employed to identify gene duplication patterns (tandem and segmental duplication), which were considered to provide genetic materials to generate new genes and support the evolutionary formation of new gene biological functions [32, 36–39]. The results showed that 137 segmental duplication events were detected for 146 *CsWRKY* genes (Fig. 5, Additional file 4: Table S4). However, no tandem duplication was observed for any *CsWRKY* gene. No tandem duplication event was further confirmed on the basis of the method by Guo et al. [40]. Compared to other tested plant *WRKY* gene families (see Additional file 5: Table S5), the *CsWRKY* genes experienced the largest number of segmental duplication events. These events revealed that segmental duplication was a major driving force for *CsWRKY* gene evolution.

**Fig. 6:**
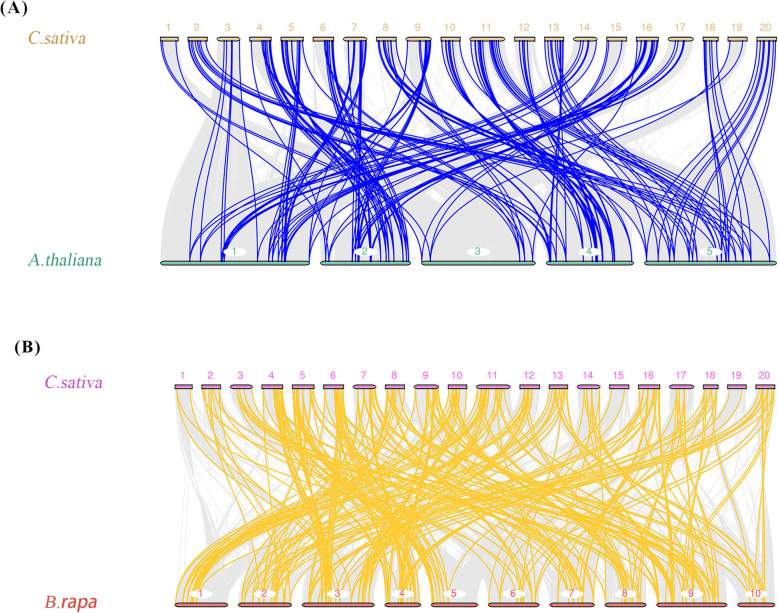
Synteny analysis of CsWRKY genes between *C. sativa* and two plant species (*A. thaliana and B. rapa*). **a** *C. sativa* and *A. thaliana* (**b**) *C. sativa* and *B. rapa*. The collinear blocks between *C. sativa* and other species was showed gray lines. The syntenic WRKY gene pairs between *C. sativa* and *A. thaliana* were highlighted with blue. The syntenic WRKY gene pairs between *C. sativa* and *B. rapa* were highlighted with yellow. The chromosome number was indicated at the top of every chromosome

The comparative synteny maps of two related genomes (*C. sativa* VS *A. thaliana*, and *C. sativa* VS *Brassica rapa* (*B. rapa*)) were created to explore the origin and evolution of *CsWRKY* genes (Fig. 6, Additional file 6: Table S6). Orthologous relationships were detected between 173 *CsWRKY* genes and 65 *AtWRKY* genes, and then 173 orthologous *WRKY* gene pairs were identified accordingly, with most of them located on the syntenic locus in *Arabidopsis* and *C. sativa* chromosomes. Similarly, the orthologous relationships were also present between 166 *CsWRKY* genes and 111 *BrWRKY* genes, and the corresponding 282 orthologous *WRKY* gene pairs were built, with many found on the syntenic locus in the chromosomes of *C. sativa* and *B. rapa*. Remarkably, multiple *CsWRKY* genes were identified as putative orthologs of a single *AtWRKY* gene. For example, *CsWRKY123, CsWRKY124 CsWRKY125* and *CsWRKY126* were the orthologs of *AtWRKY39*. This syntenic relationship detection in these *WRKY* genes indicates that the expansion of *CsWRKY* genes may have occurred after that of *A. thaliana* in evolution.

To investigate whether these orthologous *WRKY* genes underwent selection pressure (purifying and positive selection), the synonymous substitution rates (Ks) and non-synonymous substitution rates (Ka) of the identified orthologous *CsWRKY* gene pair were calculated using KaKs Calculator 2.0, followed by calculations of the Ka/Ks to determine if the selective pressure acts on protein-coding *CsWRKY* genes or not [41]. Interestingly, the Ks and Ka values for all the orthologous *CsWRKY* gene pairs had a ω value of < 1, indicating that purifying selection occurred in these gene pairs, which is consistent with the reports on *B. rapa* by Tang et al. [18].

### Expression patterns of *CsWRKY* genes in twelve camelina tissues

**Fig. 7:**
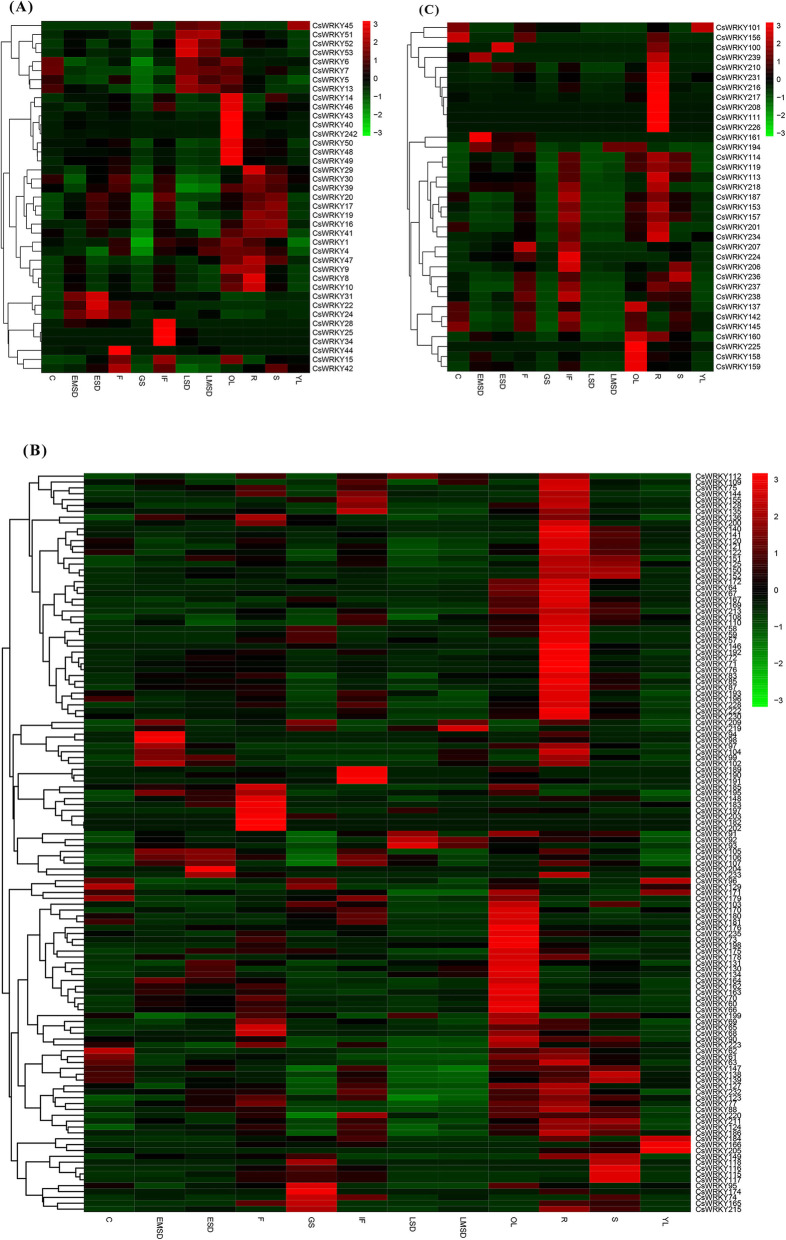
Heat map representation and hierarchical clustering of the CsWRKY gene expression profiles in twelve tissues. **a** Group 1. **b** Group 2. **c** Group 3. The expression levels of CsWRKY genes were showed by different colors on the right scale. C, cotyledon; EMSD, early-mid seed development; ESD, early seed development; F, flower; GS, germinating seed; IF, inflorescence; LSD, late seed development; LMSD, late-mid seed development; OL, mature leaf; R, root; S, stem; YL, young leaf

The gene expression pattern can provide essential information to determine the biological function of a gene. To explore the possible functions of *CsWRKY* genes in *C sativa* growth and development, transcriptome data from *C. sativa* under normal growth conditions were downloaded from the publicly available database [6] and used to examine the expression patterns of 202 *CsWRKY* genes in twelve tissues/organs, including the root (R), stem (S), young leaf (YL), mature leaf (OL), flower (F), inflorescence (IF), early seed development (ESD), early-mid seed development (EMSD), late-mid seed development (LMSD), late seed development (LSD), germinating seed (GS) and cotyledon (C). A heat map illustration of the expression profiles for the *CsWRKY* genes is shown in Fig. 7.

Notably, half more tested *CsWRKY* genes were found to be expressed in at least one of those tissues, although *CsWRKY36* and *227* did not display any detectable expression. For example, 180 CsWRKY genes (89.11%) were expressed in R while 106 CsWRKY genes (52.48%) accumulated in LSD. The percentages of expressed *CsWRKY* gene numbers were 88.61% in F, 81.68% in IF, 80.69% in S, 78.71% in OL, 77.23% in ESD, 76.73% in EMSD, 75.25% in GS, 68.32% in C, 67.82% in LMSD, and 64.85% in YL. The 70 *CsWRKY* genes (34.65%) were expressed in all twelve tissues, including 28 *CsWRKYs* in group I, 41 *CsWRKYs* in group II, and one *CsWRKY* gene in group III. In particular, 24 *CsWRKY* genes displayed high expression levels in all the tissues. Furthermore, a number of *CsWRKY* genes exhibited a tissue-specific expression pattern. For example, genes that were expressed preferentially in two tissues included *CsWRKY25* and *34* from group I in IF and LSD, *CsWRKY174* of group IIb in GS and R, *CsWRKY73* and *203* from group IIc in F and OL or GS, *CsWRKY118* from group IIe in GS and S, and *CsWRKY100* from group III in ESD and R. The genes that were specifically expressed in one tissue were *CsWRKY182* and *202* as well as *204* from group IIc in F and ESD, respectively, and *CsWRKY111, 208* and *226* from group III in R. This expression analysis indicates that most *CsWRKY* genes may act constitutively during plant organ development, with a number of them working differentially in different tissues.

### Number of *CsWRKY* genes expressed in response to various abiotic stresses

**Fig. 8:**
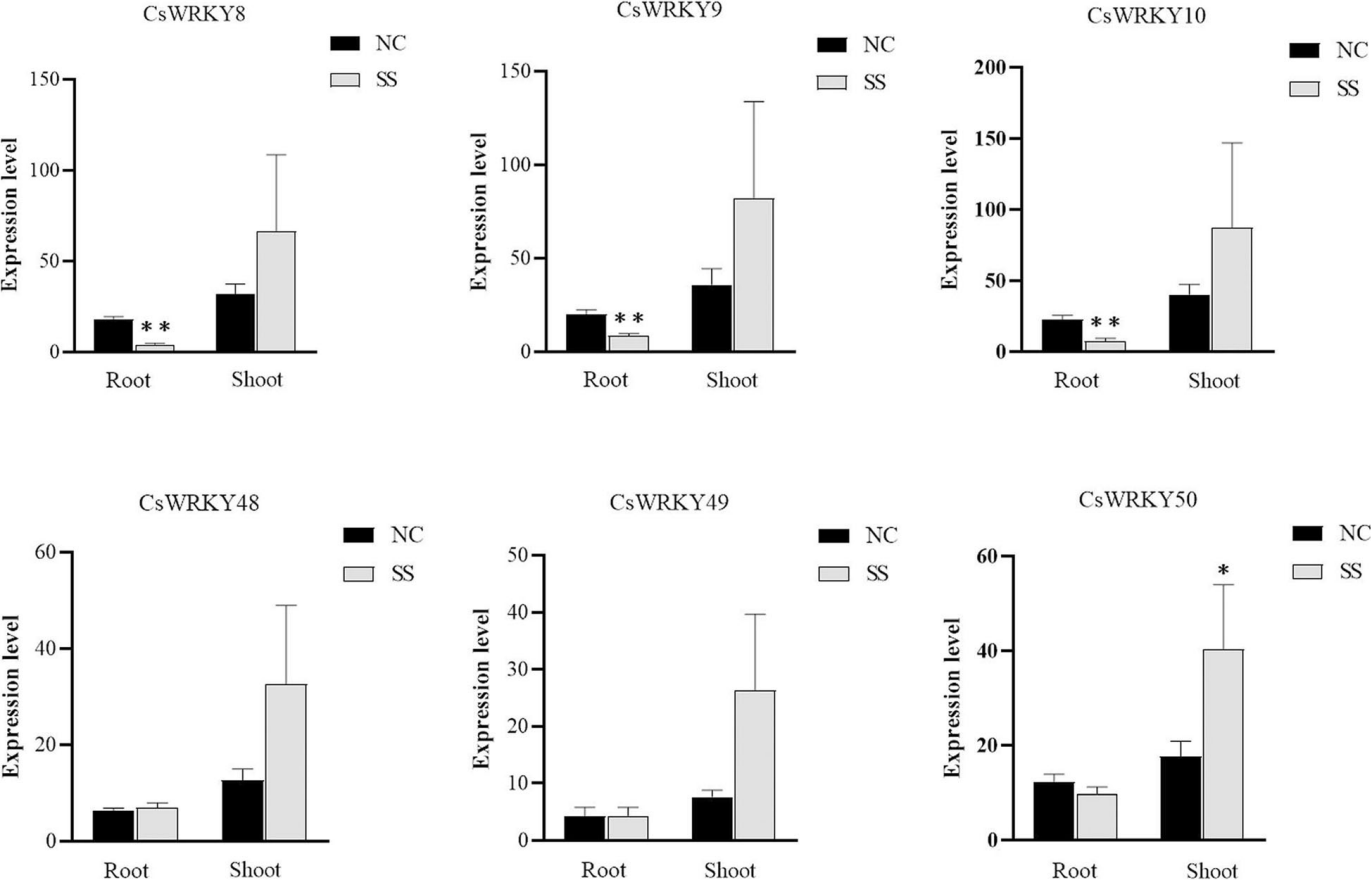
Expression profiles of CsWRKY genes in roots and shoots under salt stress. SS, salt stress. NC, normal conditions. Data are means ± SE calculated from three biological replicates. The expression levels of CsWRKY genes in different tissues were compared to NC with one-way ANOVA at significance levels of ∗∗ *P* ≤ 0.01 and ∗ *P* ≤ 0.05

The functions of most *AtWRKY* genes have been verified, which can be used to infer the potential roles of the *CsWRKY* genes clustered together with the *AtWRKYs* in the phylogenetic tree. For example, *AtWRKY25* and *33* were sensitive to NaCl stress, and overexpressing either of them enhanced NaCl tolerance in *Arabidopsis* [42]. According to the structural features and phylogenetic analysis of CsWRKYs and AtWRKYs, *AtWRKY33* was identified to be the ortholog of *CsWRKY8, 9 and 10*, while *AtWRKY25* was the ortholog of *CsWRKY48, 49,* and *50*, indicating that these six *CsWRKY* genes may participate in plant responses to NaCl stress. Subsequently, to verify this prediction, the expression profiles of the six *CsWRKY* genes were examined in the roots and shoots under salt stress (SS) and normal conditions (NC) by quantitative RT-PCR (qRT-PCR) (Fig. 8). These six genes (*CsWRKY8, 9, 10, 48, 49,* and *50*) showed higher levels of expression in the shoots than in the roots under SS. Compared with the gene expression levels in NC, the expression of three *CsWRKY* genes (*CsWRKY8, 9,* and *10*) under SS were significantly downregulated in the roots, whereas the other three *CsWRKY* genes (*CsWRKY48, 49,* and *50*) showed no obvious change in the roots. In particular, the *CsWRKY50* transcript was significantly upregulated in the shoots under SS. These results implied that the six *CsWRKY* genes may positively regulate the SS response in the shoot, whereas three of them (*CsWRKY8, 9,* and *10*) may act negatively in the roots.

**Fig. 9:**
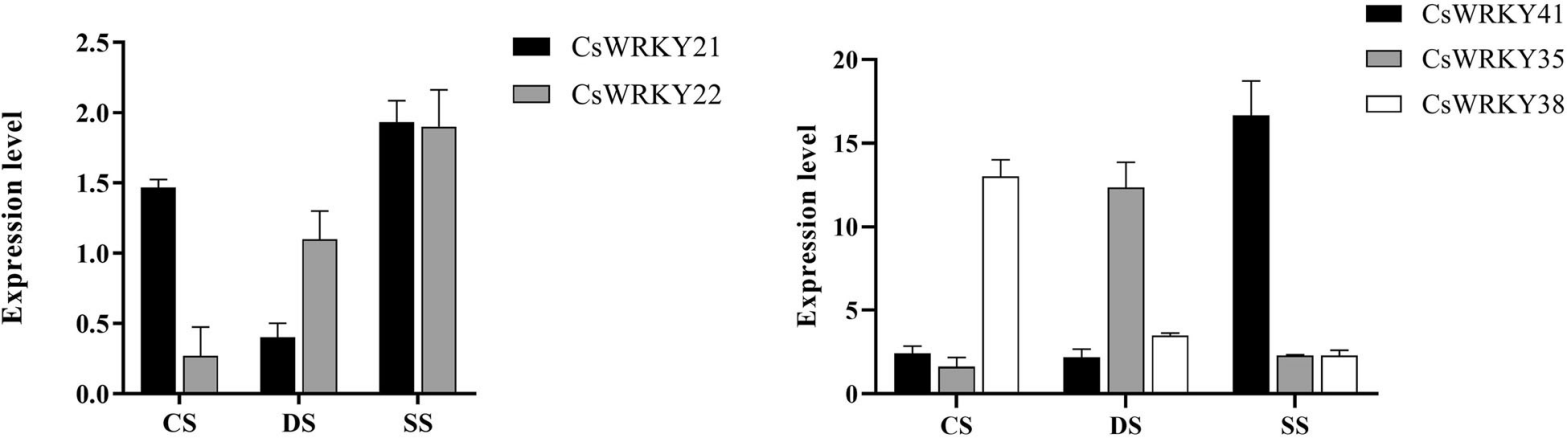
Expression profiles of different variants of CsWRKY genes in camelina seedlings under cold stress、drought stress and salt stress. CS, cold stress; DS, drought stress; SS, salt stress. Data are means ± SE calculated from three biological replicates

As described above, 15 *CsWRKY* gene loci contain alternative structures. To investigate whether all the alternative splice variants from a *CsWRKY* gene locus are involved in plant responses to various stresses, a total of 5 splice transcripts derived from two *CsWRKY* gene loci were selected to examine their expression levels in camelina seedlings treated with cold stress (CS), drought stress (DS) and, and SS, respectively. As shown in Fig. 9, these five splice isoforms showed different expressions under the three stress conditions, with three of them being expressed 10-fold higher than the other two. *CsWRKY21* and *22*, the two splice variants from gene locus *Csa06g039950*, both showed the highest expression at almost the same level under SS among the three treatments. However, *CsWRKY21* exhibited significantly greater expression under CS than under DS. By contrast, the *CsWRKY22* expression was greater under DS than under CS. Similarly, *CsWRKY35*, *38* and *41*, the three spliced variants from gene locus *Csa10g016180*, were expressed at different levels, with *CsWRKY35, 38,* and *41* being the predominant transcripts in response to DS, CS, and SS, respectively. More strikingly, these data led to unexpected findings that alternative slice variants from the same gene locus may function differentially in plant responses to different stresses.

## Discussion

### Conserved and divergent features among CsWRKY family members

As one of the largest TF families in higher plants, the WRKY family members participate in plant development and in response to various stresses. In this study, we used genome and RNA-Seq data to identify 224 *WRKY* gene loci encoding 242 CsWRKY proteins in *C. sativa*. Compared to the quantity of WRKY family members detected in other higher plants such as rice [43], soybeans [32], peppers [44], peanuts [34], and sesame [45] (see Additional file 1: Table S1 for details), the CsWRKY family is the second-large family after that of *Brassica napus* [46], with 287 WRKY members. This finding indicates that a large-scale expansion of this family occurred in the *C. sativa* genome, which might have been the result of gene duplication (see discussion below). Subsequently, these CsWRKY proteins were characterized according to the specific conserved sequences of WRKYs and the number of their WRKY zinc-finger motifs [35] according to the principle used for *Arabidopsis* [35] and common beans [47]. Based on the phylogenetic analysis of CsWRKYs and AtWRKYs, 242 CsWRKYs were divided into three primary groups: Group I, Group II (subgroup IIa, IIb, IIc, IId, and IIe) and Group III, with group II containing 149 members, which accounts for the largest proportion (62%) among all the groups. The higher proportion of group II members was also detected in other plant WRKY families, such as 58% in *Arabidopsis*, 64% in *Caragana intermedia* [48] and 66% in *Manihot esculenta* [49]. For each group, the CsWRKY members exhibited a similar structure, but members of different groups showed specific features, which is consistent with reports on other plants such as chickpeas [50] and common beans [47].

Previous reports indicated that the group I WRKY containing two WRKY domains was considered to be the most ancient member that occurred during WRKY evolution. The WRKYs in group IIa and IIb came from an algal single WRKY domain or the other Group 1-derived lineage [26, 50]. The members of group IIc evolved from the lack of N-terminal domain in the WRKYs in group II. Despite these hypotheses, the origin of each type of WRKY proteins in *C. sativa* is unknown*.*

In spite of the strong conservation of WRKY domains for WRKY proteins, CsWRKY proteins showed a certain degree of divergence in structure. The heptapeptide WRKYGQK is the typical domain for the WRKY family. However, eleven variants including WRKYGXK, WRKYGKK, and WRKYGEK for this domain were detected in a number of CsWRKY proteins in group IIc. In support of this finding, several variants of the WRKY domain were also found in in other plant species [13, 40, 51, 52], suggesting that these variants might give WRKYs multiple biological functions [47] although further research is required. In addition, we also identified several CsWRKY proteins as the chimeric proteins that contain both the R-protein conserved domain and the WRKY domain. For example, CsWRKY47, CsWRKY186 and CsWRKY220 were newly examined as R protein-WRKYs. As reported in *Arabidopsis,* AtWRKY coded an NBS-LRR-WRKY protein. This protein acted as a chimeric protein, and the WRKY domain had DNA-binding activity [53]. The interaction of AtWRKY52/RRS1 with R protein RPS4 protected the plants from infection by fungal and bacterial pathogens [39]. Possibly, the R protein-WRKYs detected in *C. sativa* can participate in plant resistance to disease or other stresses, as in the case of *Arabidopsis*, but the putative function must be further investigated.

### Segmental duplication contributed critically to the expansion of the *CsWRKY* gene family in evolution

Increasing numbers of reports have indicated that gene duplication (e.g., tandem duplication, segmental duplication, and genome duplication) was the key force for gene family expansion in plant genomes [50]. Here, the genome-wide identification and analysis of the WRKY gene family in *C. sativa* identified 137 segmental duplication events but no tandem duplication event. Furthermore, the comparative synteny map between *C. sativa* and *A. thaliana* provided more evidence for the gene duplication generated during the evolution of *C. sativa*. A total of 173 *CsWRKY* genes were identified to have 65 corresponding *Arabidopsis* orthologs, and even multiple *CsWRKY* genes were found to correspond to a single *AtWRKY* ortholog. All these data demonstrate that segmental duplication is the major force for the expansion of the *CsWRKY* gene family. As the key factor, segmental duplication was detected to be related to the expansion of the WRKY gene family in chickpeas, pineapples and kiwifruits [24, 27, 50]. Therefore, we can speculate that segmental duplication events are the primary cause driving the *CsWRKY* gene family to expand to such large members. It is notable that genome duplication, tandem duplication, and segmental duplication were combined together to form the evolution force for gene duplication in other plants, particularly diploidized polyploids. It was also reported that tandem duplication was the primary contributor to the enlargement of *BnaWRKY* genes in group III [46], and the duplication events did not occur in the pepper *WRKY* gene family [44].

The genetic evolutionary process was probably re-established by comparing all the gene sequences within the same genome or among the different genomes [54]. Notably, according to the syntenic map of *C. sativa* and *B. rapa*, 166 *CsWRKY* genes were found to have 111 corresponding *B. rapa* orthologs. Thus, the expansion of the *CsWRKY* gene family likely occurred after the separation of *A. thaliana* and *B. rapa*.

These duplicated *CsWRKY* genes probably formed new gene functions to adapt to various growth conditions. The majority of Ka/Ks ratios for the above syntenic *WRKY* gene pairs were less than 1, which demonstrated that *CsWRKY* genes underwent strong evolution by purifying selection. Purifying selection (Ka/Ks ratio < 1) has usually caused the elimination of deleterious genes by evolution and is favoured to generate genes with conserved functions, which indicated that the key conserved sequences of *WRKY* genes were eventually beneficial for plant survival and growth [7, 50].

### CsWRKY proteins may be major players in response to various stresses

In plants, the WRKY proteins act as one of the most important TF families and appear to regulate plant responses to biotic and abiotic stresses by acting as positive or negative regulators. Many *WRKY* genes were found to respond to various stress in *Arabidopsis*, rice and soybeans. For example, the high expression of WRKYs had a vital influence on the transcription activation of downstream target genes involved in plant growth and development [29]. The tissue-specific expression of WRKY genes might have great effects during plant growth and development by regulating the transcriptional process [18]. In the present study, many *CsWRKY* genes were found to be constitutively expressed in various tested tissues, with many of them showing a tissue-specific expression pattern. More importantly, a number of *CsWRKY* genes exhibited a dramatic expression change under various stress conditions. Six of the *CsWRKY* genes (*CsWRKY8, 9, 10, 48, 49,* and *50*) were further selected for investigation, indicating that they positively regulate the stress responses in shoots, but *CsWRKY8, 9,* and *10* play negative roles in the roots. Transgenic experiments are currently under way in *C. sativa* to identify their precise biological functions and to evaluate their potential use in genetic engineering for improving crop stress resistance and other agronomic traits.

One of the novel findings in this study is that five splice isoforms (*CsWRKY 21, 22, 35, 38* and *41*) derived from two *CsWRKY* gene loci were differently expressed in plant seedlings under cold, salt, and drought stresses. In view of this finding, we can speculate that different alternative variants from the same gene locus function differentially in different stress responses. However, the accurate regulatory mechanism must be elucidated in further detail.

## Conclusion

In this study, a total of 242 CsWRKY proteins were identified for the first time in *Camelina sativa*, and they were classified into three primary groups (I, II, and III). There were 33 alternative splicing events detected for 15 *CsWRKY* gene loci. Many orthologous *WRKY* gene pairs were identified between every two genomes of *C. sativa*, *A. thaliana*, and *B. napus,* showing a high synteny among the three genomes. Segmental duplication events were found to be the major force of great expansion by the *CsWRKY* gene family. Moreover, the camelina WRKY proteins experienced a strong purifying pressure over their evolution. Half more *CsWRKY* genes were expressed in various organs, with many *CsWRKYs* having a tissue-specific expression pattern, demonstrating that CsWRKYs have critical functions in plant development and also act differentially in different tissues. Remarkably, eleven CsWRKYs, including five alternative spliced isoforms, may play crucial roles in regulating plant responses to various stresses, although this interpretation will require extensive additional analyses. Overall, our study establishes a functional framework to investigate CsWRKY proteins and the mechanisms responsible for high resistance to various stresses*,* facilitating the development of molecular breeding programs to enhance abiotic stress tolerance in camelinas and other crops.

## Methods

### Sequence identification

The complete genome, proteome and CDS sequence files of *C. sativa* were downloaded from the webpage of the NCBI (ftp://ftp.ncbi.nlm.nih.gov/genomes/all/GCF/000/633/955/GCF_000633955.1_Cs/) and Genome Prairie - Prairie Gold (http://www.camelinadb.ca). WRKY domain HMM (Hidden Markov Model) profile numbered PF03106 was extracted from the Pfam protein family database (http://pfam.xfam.org/family/PF03106#tabview=tab6) [47]. The candidate WRKY protein sequences were discovered by comprehensive research using HMMER (E-value cut-off <1E-5) and BLAST analyses (72 AtWRKYs as queries) in the *C. sativa* whole genome protein database [49]. The AtWRKYs sequences were obtained from the NCBI (https://www.ncbi.nlm.nih.gov/genome/?term=Arabidopsis±thaliana). These CsWRKYs sequences were identified by checking the complete WRKY conserved domain with SMART (http://smart.embl-heidelberg.de/) and InterPro (http://www.ebi.ac.uk/interpro/), and the redundant sequences were manually removed. The confirmed CsWRKYs employed the ExPasy online tool website (http://web.expasy.org/protparam/) to calculate the physicochemical properties, including the SL, MW and PI.

### Multiple sequence alignment and the construction of the comparative phylogenetic tree

A multiple sequence alignment of the CsWRKY domain was performed using ClustalW with Bioedit software. Based on the alignment of the WRKY domains of CsWRKYs and AtWRKYs, a phylogenetic tree was constructed with MEGA 7.0 using the neighbour-joining method and the relevant parameters (Poisson model, pairwise deletion, and 1000 bootstrap replications). All the identified CsWRKYs were divided into different groups according to the classification of AtWRKYs sequences.

### Motif analysis

MEME (http://meme.nbcr.net/meme/intro.html) was used to analyse the CsWRKYs and searched for 10 conserved motifs. The interrelated parameters were as follows: the repetitive time was any, the maximum motif number was 20 and the motif width was between 5 and 50 residues. The MEME results were displayed with TBtool software [55].

### Chromosomal distribution

The chromosomal positions of all the CsWRKY genes were determined from the genome annotation file. The physical position of the CsWRKY genes from the short-arm to long-arm telomeres on the chromosome was mapped using MapChart [56].

### Gene duplication and selection pressure analyses

Segmental duplication events within the *C. sativa* genome were studied by using MCScanX (Multiple Collinearity Scan toolkit), and the entire analytic process was used in the default setting. The tandem duplication event (the distance of two or more genes within 200 kb in equal chromosomes) was checked on the basis of the method by Guo et al. [40]. The synteny relations between two different genomes (*C. sativa* and *A. thaliana*, *C. sativa* and *B. rapa*) were identified and analysed. The *B. rapa* data were downloaded from the EnsemblPlants database (https://plants.ensembl.org/*Brassica rapa*/Info/Index). The Ks and Ka of the identified CsWRKY gene pair were calculated using KaKs Calculator 2.0 [41].

### Expression profiling of CsWRKY genes in different tissues

The transcriptional data of *C. sativa* was obtained from a publicly available database [6]. In twelve tissues at various developmental stages, the expression levels of the CsWRKY genes were used for the analysis, including C, EMSD, ESD, F, GS, IF, LSD, LMSD, OL, R, S and YL. The hierarchical clustering and the heatmap-based expression profiles of the CsWRKY genes were performed by HemI1.0. To explore the function of the CsWRKY genes, we researched whether some CsWRKY genes respond to SS. The RNA-Seq data from *C. sativa* that we used in response to SS came from a publicly available database [4]. *C. sativa* cultivar “SC-N1”, which was commercially planted for 5 years in Taigu County, Shanxi province, China (E112.32°, N37.26°), was selected for further experiments [5]. The plants were grown in a greenhouse (16-h light/8-h dark, 23 °C). The 3-week-old soil-grown seedlings were treated using cold stress (CS), drought stress (DS) and SS. The seedlings were treated at 4 °C for CS. The soil moisture content was 35% ± 5 of NC for DS. The seedlings were irrigated with 150 mM NaCl solutions in climatic chambers. The seedlings were treated for up to 3 days. These camelina seedlings were collected and frozen immediately in liquid N_2_ for RNA extraction at 3 days after the different treatments. A TaKaRa RNAiso Plus kit was used to extract the total RNA from all the samples. The 1 μg RNA was reverse-transcribed into cDNA with a TaKaRa reverse transcription kit, and the same amount of cDNA was used as a template for qRT-PCR. The β-actin gene of *C. sativa* was selected as the internal reference gene [5]. All the primer pairs used for qRT-PCR analysis are shown in Additional file 7: Table S7. These experiments were repeated three times.

## Supplementary Information

**Additional file 1 **: **Table S1**. The number of WRKY genes in the reported species.

**Additional file 2 **: **Table S2**. Detailed information of all identified *Camelina sativa* WRKY proteins.

**Additional file 3 **: **Table S3**. Alternative splicing of WRKY genes.

**Additional file 4 **: **Table S4**. Segmental duplication of CsWRKY among *C. sativa* chromosomes.

**Additional file 5 **: **Table S5**. Tandem duplication and segmental duplication of some species.

**Additional file 6 **: **Table S6**. All gene pairs of two different genomes (*C. sativa* and *A. thaliana*, *C. sativa* and *B. rapa*).

**Additional file 7 **: **Table S7**. Primer pairs used for qRT-PCR analysis on the target sequences in *C. sativa.*

**Additional file 8 **: **Figure S1**. Schematic representation of splice pattern of some CsWRKY genes.

**Additional file 9 **: **Figure S2**. Multiple sequence alignment of all CsWRKYs.

**Additional file 10 **: **Figure S3**. Phylogenetic tree (left) and motif distributions (right) of the CsWRKY proteins.

**Additional file 11 **: **Table S8**. All accession numbers of sequences used in article.

## Data Availability

All the data generated or analysed during this study are included in this published article and its supplementary information files (from Additional file 1 to Additional file 11). The WRKY domain HMM (Hidden Markov Model) profile numbered PF03106 was extracted from the Pfam protein family database (http://pfam.xfam.org/family/PF03106#tabview=tab6). The complete proteome and CDS sequence files of *C. sativa* were downloaded from the NCBI webpage (ftp://ftp.ncbi.nlm.nih.gov/genomes/all/GCF/000/633/955/GCF_000633955.1_Cs/). The complete genome sequence file of *C. sativa* was downloaded from Genome Prairie - Prairie Gold (http://www.camelinadb.ca). The AtWRKY sequences were obtained from the NCBI (https://www.ncbi.nlm.nih.gov/genome/?term=Arabidopsis±thaliana). The *B. rapa* data were downloaded from the EnsemblPlants database (https://plants.ensembl.org/Brassica rapa/Info/Index). All accession numbers of sequences used in article were showed in Additional file 11: Table S8.

## Abbreviations

WRKYs: WRKY proteins
TF: Transcriptional factor
qRT-PCR: Quantitative Real-time RT-PCR
AS: Alternative splicing
SL: Protein sequence length
MW: Molecular weight
PI: Isoelectric point
Ks: Synonymous substitution rates
Ka: Non-synonymous substitution rates
*C. sativa*: *Camelina sativa*
*A. thaliana*: *Arabidopsis thaliana*
*B. rapa*: *Brassica rapa*
C: Cotyledon
EMSD: Early-mid seed development
ESD: Early seed development
F: Flower
GS: Germinating seed
IF: Inflorescence
LSD: Late seed development
LMSD: Late-mid seed development
OL: Mature leaf
R: Root
S: Stem
YL: Young leaf
SS: Salt stress
NC: Normal conditions
CS: Cold stress
DS: Drought stress
