# Supplementary figures and images for: Genome-wide identification and functional characterization of the Camelina sativa WRKY gene family in response to abiotic stress
Source: BMC Genomics. 2020 Nov 11;21:786. doi: 10.1186/s12864-020-07189-3 (PMC7659147; doi:10.1186/s12864-020-07189-3)

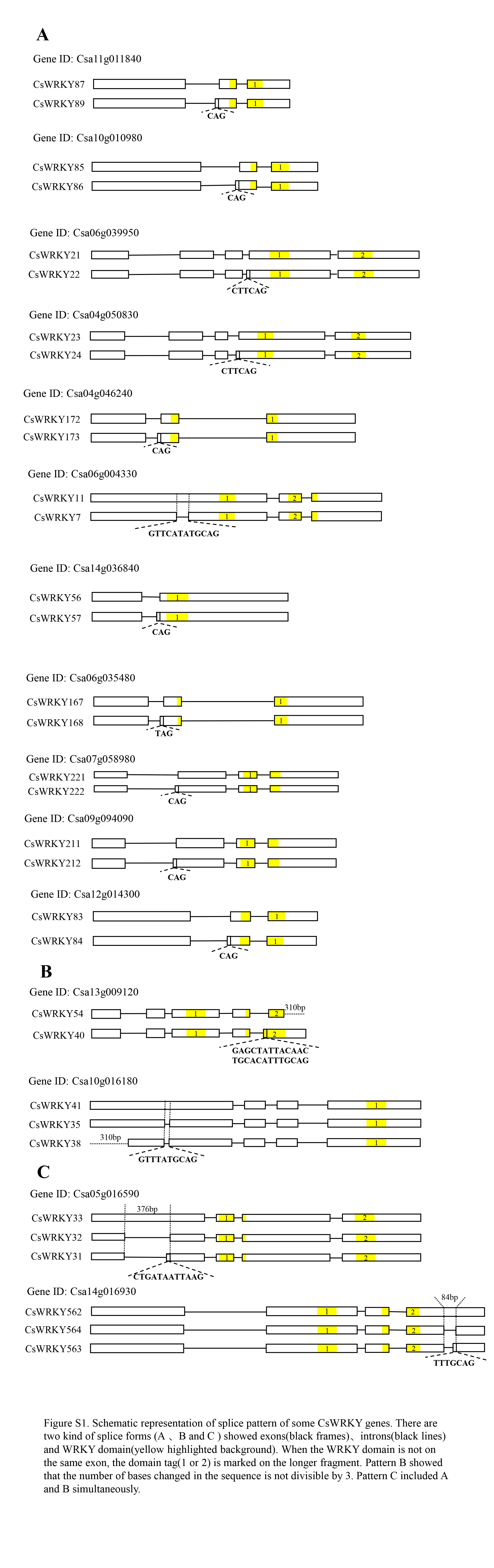

Supplement: Supplementary file 8 — Additional file 8 : Figure S1. Schematic representation of splice pattern of some CsWRKY genes. [file 12864_2020_7189_MOESM8_ESM.jpg]

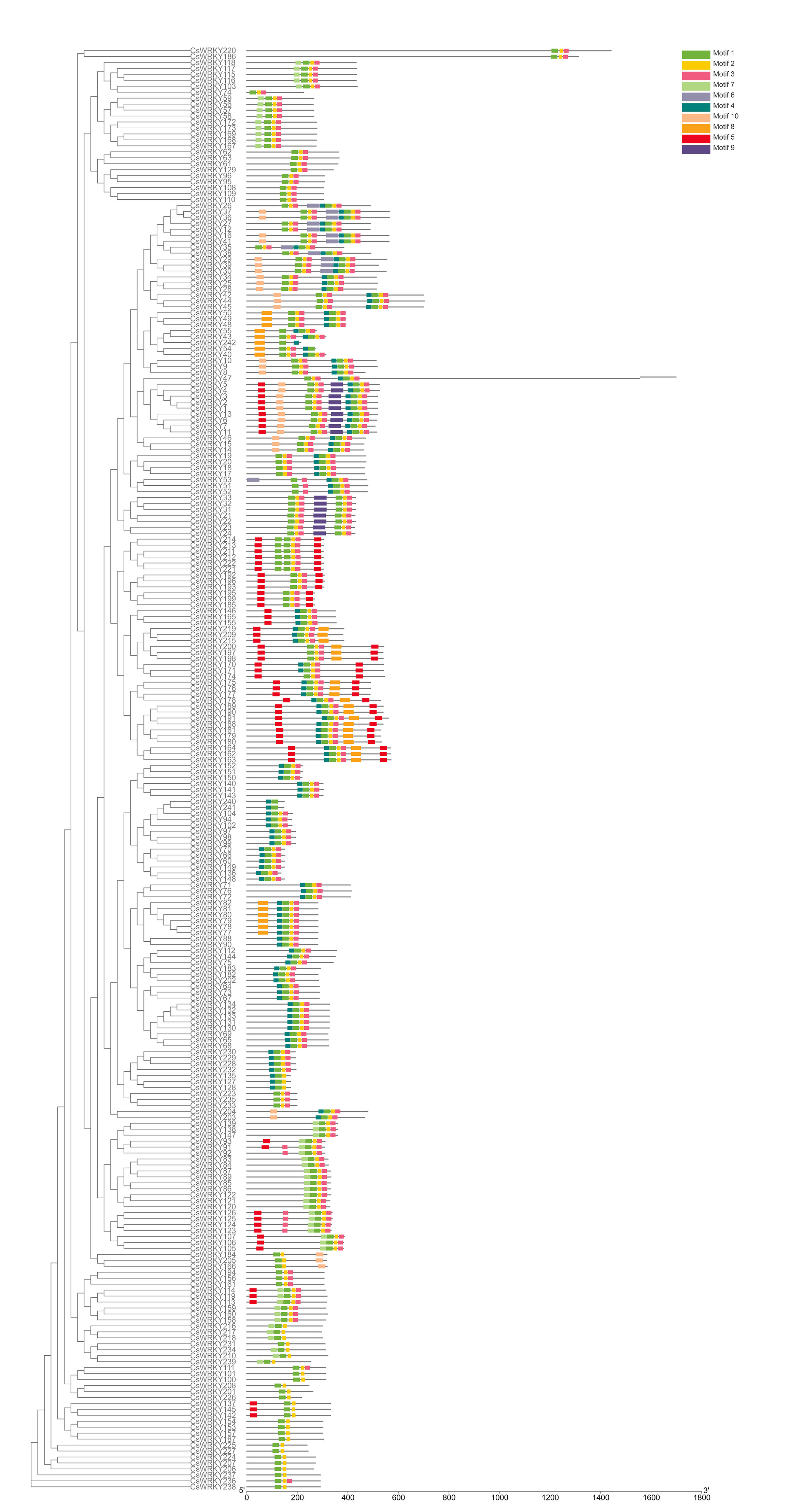

Supplement: Supplementary file 10 — Additional file 10 : Figure S3. Phylogenetic tree (left) and motif distributions (right) of the CsWRKY proteins. [file 12864_2020_7189_MOESM10_ESM.jpg]
